# Passive Electroluminescence and Photoluminescence Imaging Acquisition of Photovoltaic Modules

**DOI:** 10.3390/s24051539

**Published:** 2024-02-28

**Authors:** Alberto Redondo-Plaza, José Ignacio Morales-Aragonés, Sara Gallardo-Saavedra, Héctor Felipe Mateo-Romero, Santiago Araujo-Rendón, Ángel L. Zorita-Lamadrid, Víctor Alonso-Gómez, Luis Hernández-Callejo

**Affiliations:** 1 Department of Agricultural and Forestry Engineering, University of Valladolid, 42004 Soria, Spain; sara.gallardo@uva.es; 2 Department of Applied Physics, University of Valladolid, 47002 Valladolid, Spain; joseignacio.morales@uva.es (J.I.M.-A.); victor.alonso.gomez@uva.es (V.A.-G.); 3 Department of Condensed Matter Physics, University of Valladolid, 47002 Valladolid, Spain; hectorfelipe.mateo@uva.es; 4 Department of Applied Mathematics, Science and Engineering of Materials, and Electronic Technology, Rey Juan Carlos University, 28933 Madrid, Spain; s.araujo.2020@alumnos.urjc.es; 5 Department of Electrical Engineering, Universidad de Valladolid, 47002 Valladolid, Spain; zorita@uva.es

**Keywords:** renewable energy, solar energy, photovoltaic, inspection techniques, luminescence, electroluminescence, photoluminescence, passive luminescence

## Abstract

In photovoltaic power plant inspections, techniques for module assessment play a crucial role as they enhance fault detection and module characterization. One valuable technique is luminescence. The present paper introduces a novel technique termed passive luminescence. It enhances both electroluminescence and photoluminescence imaging acquisition in photovoltaic power plants under normal operation in high irradiance conditions. This technique is based on the development of an electronic board, which allows the polarity of the module to be changed, enabling the current generated by the photovoltaic string to be injected into the module and producing electroluminescence effects. Additionally, the board can bypass the module and set an open circuit, inducing photoluminescence emission using sunlight as an excitation source. The proper coordination of the board and an InGaAs camera with a bandpass filter has allowed for the integration of a *lock-in* technique, which has produced electroluminescence and photoluminescence pictures that can be used for fault detection.

## 1. Introduction

The global economy and society necessitate a significant transformation to mitigate greenhouse gas emissions and curb the impacts of global warming. Notably, the energy sector emerges as a primary contributor, accounting for 65% of total greenhouse gas emissions [1]. Within the energy sector, 42% of these emissions are attributed to the combustion of fossil fuels for electricity and heat generation [1], underscoring the imperative for the electricity industry to align with environmental objectives set by the Paris Agreement [2]. The adoption of renewable electricity generation with a low carbon footprint is pivotal for achieving the decarbonization of the electrical sector. As of 2022, 30% of the total global electricity is derived from renewable sources. Hydropower constitutes the majority at 15.0% of the total global electricity, followed by wind energy at 7.3% and solar energy at 4.6% [3].

Photovoltaic (PV) power has experienced substantial growth in recent years, increasing for a total global capacity of 39.3 GWp in 2010 to current 1061.7 GWp in 2022 [3]. Additionally, forecasts indicate that PV power will convert the technology for electricity generation with highest installed global capacity by 2027, surpassing a total of 2300 GWp [4]. The growth of PV systems is mainly explained by the cost reduction of the PV panel, the most expensive part of any PV system within its installation and operation. This cost reduction has led to an average levelized cost of energy (LCOE) for utility PV power plants around 40 USD/MWh [5], which makes energy generated by PV the cheapest way to produce electricity.

Maintenance plays a crucial role in the effective operation of a PV power plant [6,7,8], enabling the maximization of energy generation and preventing safety issues. Among the various maintenance tasks essential for optimal performance in PV power plants, the inspection and characterization of solar modules are particularly critical. PV modules are exposed to a variety of environmental loads, including mechanical loads, thermal loads, ultraviolet radiation, wind loads, moisture, corrosion, etc. These factors can contribute to the occurrence of faults, resulting in a reduction of energy output and an increase in safety risks. Hence, inspection techniques such as visual inspections, current–voltage curve measurements, infrared thermography imaging, and luminescence imaging are imperative for assessing the condition of PV modules [9]. These methods are essential for identifying potential issues and ensuring the overall health and safety of the PV system.

Luminescence imaging relies on capturing the electromagnetic emission produced by crystalline structures that make up the solar cells when exposed to specific energy sources. This phenomenon can manifest in two distinct ways. Firstly, luminescence can be generated within a PV device when current is injected, a phenomenon known as electroluminescence (EL). Alternatively, luminescence effects can also occur when the PV device is exposed to light of a certain wavelength and intensity, termed as photoluminescence (PL). The wavelength of luminescence is contingent upon the material used in the construction of the solar cell. In the case of most commercial modules, which predominantly employ crystalline silicon and constitute 95% of the market [10], the luminescence wavelength peak is typically around 1150 nm [11]. It is important to note that other technologies may exhibit varying luminescence wavelengths.

This electromagnetic emission can be captured using primarily two types of cameras: those based on silicon sensors and those based on InGaAs sensors [11]. Silicon sensors are cost-effective and offer high resolution. However, their sensitivity to the silicon luminescence wavelength is low, necessitating a completely dark environment and extended exposure times. On the other hand, InGaAs sensors exhibit sensitivity that aligns with silicon luminescence emission, allowing for reduced exposure times and facilitating acquisition under non-null irradiance conditions. Nevertheless, InGaAs sensors typically have lower resolution and a higher price in comparison.

Luminescence images provide a powerful tool for detecting various faults, including finger failures, potential-induced degradation (PID), short-circuit or open-circuit faults in bypass diodes, damages induced by mechanical loads, corrosion due to moisture, shunt faults, faults related to series resistance, and the presence of cracks or microcracks [9]. In addition, luminescence images not only offer a qualitative means of detecting and analyzing failures in PV cells or modules but also prove valuable for quantitative analysis. Recent approaches have demonstrated the utility of luminescence images in quantifying various aspects of the module, such as determining the individual operational voltage of each cell within a module [12], establishing the minority carrier lifetime [13], or constructing series and shunt images for one-diode model parameters extraction and power predictions [14].

Luminescence images offer additional and highly valuable information compared to other inspection techniques such as current-voltage curves of thermography inspections [15], as luminescence unveils the behavior of the inner structure of the cell. Both EL and PL techniques have become standard methods in laboratories and factories worldwide. However, despite their significant utility, the application of these techniques for field measurements in PV power plants is limited. The intensity of the luminescence signal is two or three orders of magnitude lower than that of sunlight, making it challenging to acquire images under high irradiance conditions. Consequently, luminescence images in PV power plants have traditionally been captured during the night.

The conventional technique involves using a power supply for current injection into the PV array to produce the EL effect, and the signal is then captured with a camera during the night. However, this method has some drawbacks. Firstly, capturing pictures during the night is undesirable due to operational and safety concerns. Secondly, the technique necessitates disconnections of solar modules, increasing the time required for measurements and introducing safety concerns. Therefore, there is a need for the development of new techniques to establish luminescence imaging as a standard method in PV power plants.

In recent years, several new luminescence techniques have been developed. Table 1 provides a summary of all the approaches enabling luminescence imaging acquisition without resorting to conventional techniques. It is worth noting that the *lock-in* technique has become a standard in luminescence imaging acquisition under high irradiance conditions. The *lock-in* technique involves acquiring pairs of images where the luminescence signal is high and null, respectively. The subtraction of these images helps eliminate the background signal generated by sunlight. Given that the background signal significantly surpasses the luminescence signal intensity, this process needs to be repeated multiple times to obtain a picture of sufficient quality for fault detection. Therefore, the *lock-in* technique requires modulating an EL or PL signal in the tested PV module. The modulation can be accomplished through various methods, leading to different techniques.

The integration of unmanned aerial vehicles (UAVs) and luminescence image acquisition has become an intriguing prospect as it allows for the automation of field inspections and measuring time reduction. The use of InGaAs-based sensors is imperative to minimize exposure time and facilitate EL and PL imaging [29]. EL measurements with UAV integration have been achieved during the night using both conventional techniques [30] and bidirectional inverter-based techniques [31]. Furthermore, luminescence inspections with UAVs are feasible during the day through the utilization of a *lock-in* technique [32]. However, this integration poses challenges due to the necessary stability, necessitating special post-processing that includes edge detection and motion compensation in the images.

The present paper introduces a novel approach, which has been termed passive luminescence. The term “*passive luminescence*” refers to the technique’s ability to obtain both EL and PL while the solar power plant is in operation, resulting in a non-invasive technique that does not require an external energy input thanks to the use of an electronic device. This represents a novelty and milestone in terms of techniques for acquiring EL and PL images in photovoltaic power plants. The method involves an electronic board connected within a PV string, capable of switching the polarity of the module to induce EL effect using the current generated by the remaining modules of the string. Additionally, the electronic board can bypass the module, setting it to an open circuit state, and thus generate the PL effect using sunlight as the excitation source. Consequently, it becomes possible to integrate a *lock-in* technique for both EL and PL image acquisition.

Subsequently, the structure of the article is explained. The second part of this paper discusses theoretical considerations to aid in understanding the technique, while the materials and methods employed are detailed in the third section. The fourth section presents the results, and the final section includes discussions and conclusions.

## 2. Theoretical Framework

The luminescence signal in a PV device is associated with its operational voltage along its current–voltage curve, and it can be estimated using the following exponential equation:

(1) $$I_{L}=C\cdot \exp\left(\frac{qV}{kT}\right)$$

where *I_L_* denotes the luminescence signal intensity and *C* is a proportionality constant dependent on the PV device and camera characteristics and configuration. Additionally, *q* represents the elementary charge, *T* denotes temperature, *k* is the Boltzmann constant, and *V* is the operational voltage of the solar cell or module.

**Figure 1:**
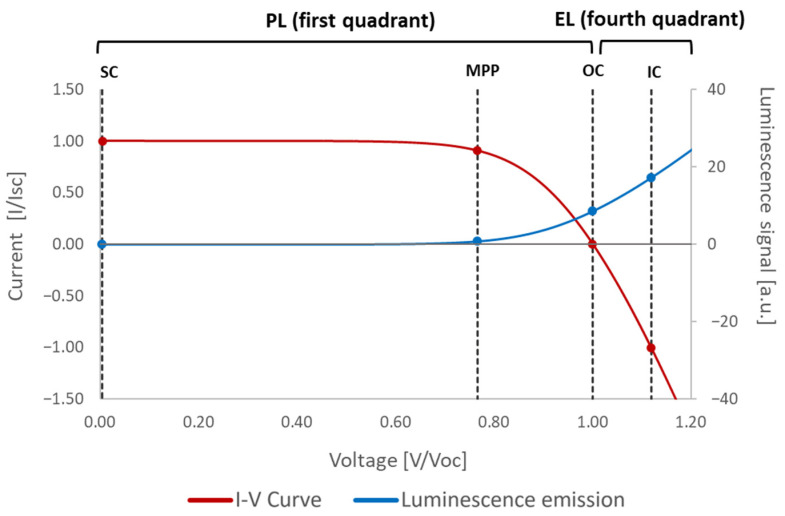
Luminescence emission thought the current–voltage curve of a PV cell or module.

Figure 1 displays a typical illuminated current–voltage curve of a PV cell or module and the variation in luminescence signal intensity depending on the working point of the curve. The PL effect is observed when the cell or module is operating in the first quadrant of the curve. In this scenario, the device functions as a generator, and sunlight produces the PL effect. This effect is null in short-circuit (SC) conditions and exponentially increases with the operational voltage. At the maximum power point (MPP), the signal is almost null, and the maximum PL signal is observed when the PV device is in open-circuit (OC) conditions. On the other hand, the EL effect requires a higher voltage than the open-circuit (OC) voltage, resulting in current injection (CI). In this case, the device operates in the fourth quadrant of the curve as a load, and PL and EL signals are added (only if the PV device is under illumination). This results in a higher luminescence intensity, which also increases with the voltage.

It should be noted that the *lock-in* technique leverages this phenomenon. As mentioned in the introduction section, the *lock-in* technique requires pairs of images where the luminescence signal is high and null or nearly null for the subtraction of images. To achieve this, it is necessary to alter the operating point of the PV cell or module along its current–voltage curve. For PL integration with the *lock-in* technique, images displaying a high luminescence signal are obtained under OC conditions, and images with a low luminescence signal are taken under MPP or SC conditions. For EL acquisition with the *lock-in* technique, images representing a high luminescence signal are captured under CI conditions, while images with a low luminescence signal are taken under MPP or SC.

## 3. Materials and Methods

**Figure 2:**
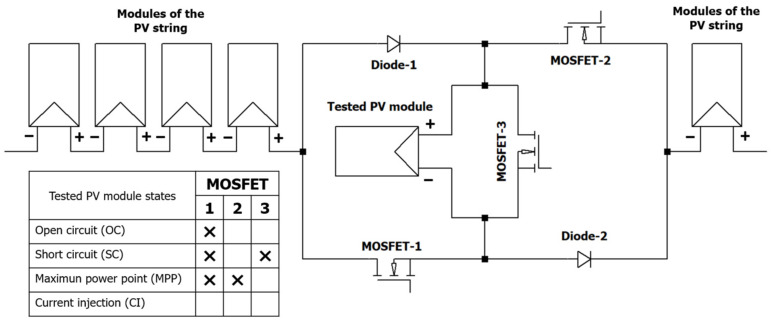
Topology of the electronic board development: MOSFETs that must be conducting to achieve OC, SC, MPP, and CI in the tested PV module are indicated with crosses in the complementary truth table.

The developed electronic device has the capability to modulate both EL and PL signals, facilitating the integration of a *lock-in* technique for imaging acquisition. Figure 2 illustrates the power topology of the electronic board, which is based on three metal-oxide-semiconductor field-effect transistors (MOSFETs) and two diodes. The electronic board can be connected within a PV string and can operate while the PV power plant is in operation. In this scenario, the state of the tested module is at the MPP, as set by the inverter. For CI, the electronic board changes the polarity of the module, allowing the current generated by the remaining modules in the string to be injected into the tested PV module, achieving the EL effect. Through the control of the MOSFETs, the board is also capable of bypassing the module and shifting its working point between open-circuit (OC) and short-circuit (SC) conditions for PL imaging acquisition with *lock-in*. Figure 2 also shows a truth table, which indicates with a cross the MOSFETs that must be under conductions to achieve the different states required.

The topology of the electronic board has been validated in LTspice 17.1.6.0 [33], utilizing the one-diode model [34] to simulate the electrical behavior of a PV module. Note that the behavior of the PV inverter could not been simulated, so the PV string has been simulated connected to a fixed charge with a certain value which sets MPP in the current–voltage curve.

**Figure 3:**
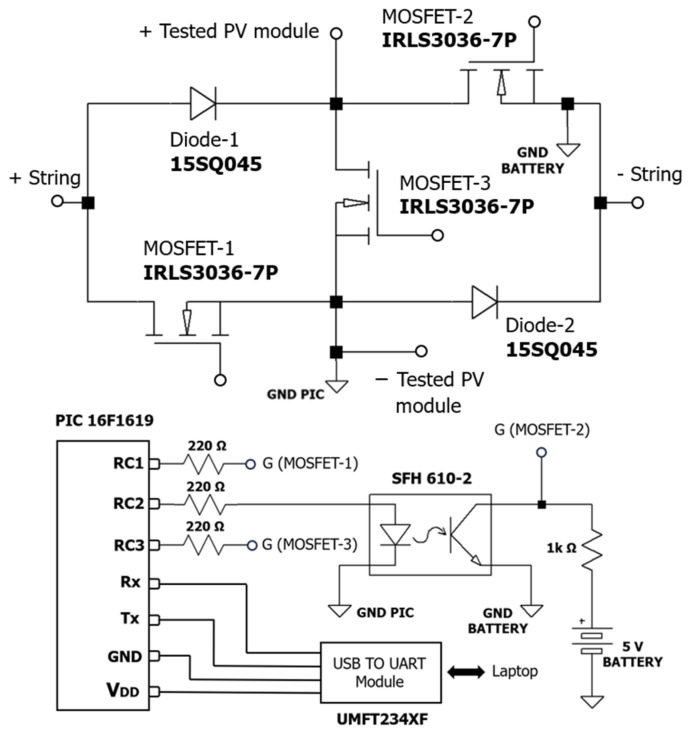
Electronic design of the developed board.

The simulation process was followed by the construction of a prototype board for validation in a real PV power plant. All the components used in the construction of the board and the designed circuit can be seen in Figure 3. Note that MOSFET-2 has its source connected to a different node than MOSFET-1 and MOSFET-3. Therefore, it cannot be directly controlled by one of the microcontroller pins. Instead, an external 5 V battery and an optocoupler have been implemented to control this transistor. The microcontroller has been programmed to receive commands through its serial port to set the module in different required states (OC, SC, MPP, and CI).

**Figure 4:**
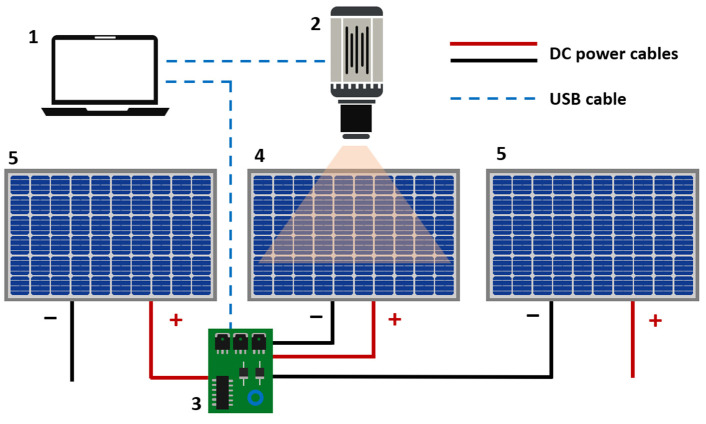
Set up for passive luminescence imaging acquisition: 1 (laptop), 2 (InGaAs camera), 3 (developed electronic board), 4 (tested module), and 5 (rest modules of the string).

The previous board has been validated in a real PV power plant. Figure 4 displays the setup used to acquire passive luminescence during daylight conditions. The developed electronic device has been connected within a string comprised of ten healthy 235 Wp polycrystalline PV modules (TSM-235-PC05A, Trina Solar Limited, Changzhou, China) that have been operating for a few years. It should be noted that each module requires one electronic board to be coupled to perform measurements in all the modules of the string. The string is connected to a string solar inverter, which is also connected to the grid. A laptop is connected to the board and to an InGaAs camera C12741-03, Hamamatsu Photonic K.K., Hamamatsu, Japan). A custom bandpass filter (Álava Ingenierios S.A., L’Hospitalet de Llobregat, Spain) has also been used, which allows blocking light with higher and lower wavelengths than luminescence emission. This is quite important as it enhances the maximization of the percentage of luminescence signal intensity compared to background signal.

A Python script has been developed to synchronize the image acquisition with the electronic board. Therefore, it is possible to obtain an image stack that contains alternate images where the luminescence signal is high and null, respectively. To maximize quality, it has been crucial to alternate between capturing pictures when the luminescence signal is high and low, rather than capturing all pictures under a high signal state followed by capturing all pictures under a low signal state. This chosen approach minimizes measurement errors caused by changes in irradiance because the shorter the time between high and low luminescence signal pictures, the more similar the irradiance between them. For proper subtraction of pictures to remove background signal, it is desirable to have the most similar irradiance.

**Figure 5:**
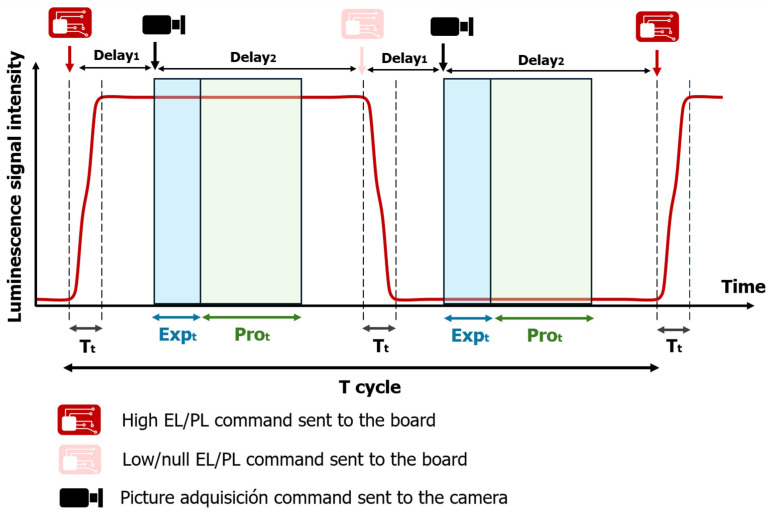
Timeline of the measurement process for one cycle of picture acquisitions. The red solid line represents luminescence signal intensity, dashed lines indicate the beginning and end of the transient time. The blue arrowhead line indicates the exposure time of the camera, and the green arrowhead denotes the required processing imaging time in the camera.

To properly synchronize the image acquisition, some issues must be considered. One cycle of pictures acquisition is shown in Figure 5. On one hand, it must be considered that there is a transient time (T_t_) between when a command is sent to the board and when the tested module changes its state. Therefore, a delay time (delay_1_) should be added between the time when the command is sent to the board and when the command is sent to the camera to acquire a picture. This transient period has been measured, and in the current setup, it is always under 0.1 ms. On the other hand, it is necessary that the tested module remains in a high or null luminescence state during the exposure time (Exp_t_) set in the camera to achieve a good measurement. Therefore, a delay time (delay_2_) should be considered to add between the time when the command is sent to the camera and the time when a command is sent to the board in order to change its state. The camera also requires a certain time to process the picture (Pro_t_), so the time required to acquire a picture (Expt + Prot) must be lower than the time between when two consecutive commands are sent to the camera. As Figure 5 shows, during one cycle, two pictures are acquired; one corresponds to a high EL/PL signal and the other corresponds to null/low EL/PL signal. In the presented work, the implemented camera requires 17 ms to process the picture. The period of the cycle (T cycle) of the current setup is around 250 ms, so a total of 8 pictures are taken per second. After the measurement process, the script set MPP in the board, so the string recovers its normal performance.

Another python script has been developed to postprocess the resulting image stack. It adds all the images corresponding to high EL or PL signal and subtracts images with null EL or PL signal to obtain a final luminescence image, which can be used as a tool for fault detection.

## 4. Results

As previously stated, the research process has been based on two steps: the simulation process and field implementation. The results of the simulation have allowed for the proper design of the topology of the board, while the implementation of the electronic board has succeeded in validating it under real conditions.

### 4.1. Simulation Validation

The simulation of the electronic device and its behavior within a PV string has enhanced the analysis of the module working point changes. Through the appropriate control of the MOSFETs, it is possible to change the working point in the module in order to generate a pulsed EL or PL signal.

**Figure 6:**
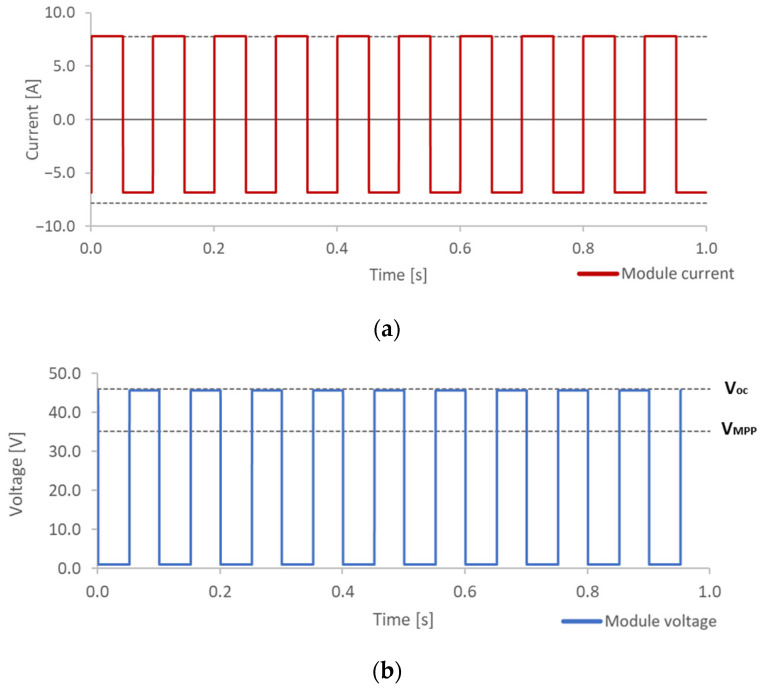
Module current evolution for pulsed EL signal generation (**a**) and module voltage evolution for pulsed PL signal generation (**b**).

On the one hand, for EL imaging acquisition, the electronic board must repetitively change the state of the tested module between CI and MPP. The evolution of the current module while it happens is shown in the first plot of Figure 6a. It is possible to see how the current changes between the MPP current, which is positive, and a negative current, which means CI into the module and therefore a high EL signal.

On the other hand, for PL imaging acquisition, the electronic board must repetitively change the state of the tested module between OC and SC. The evolution of the voltage module while it happens is shown in the second plot of Figure 6b. It is possible to see how the voltage changes between OC voltage where PL signal is high and a voltage close to 0 V, where PL signal is null.

### 4.2. Field Validation

The following measurements have been taken during a sunny day with an irradiance of around 800 W/m^2^. A proper exposure time has been set in order to make the most of the entire bit depth of the camera. It is worth noting that the intensity of the luminescence signal during this measurement process corresponds to 0.2–0.4% of the total signal captured by the camera. Therefore, several cycles of acquisition are required. In this case, 200 cycles are sufficient to obtain luminescence pictures, resulting in a total acquisition time of around 50 s.

**Figure 7:**
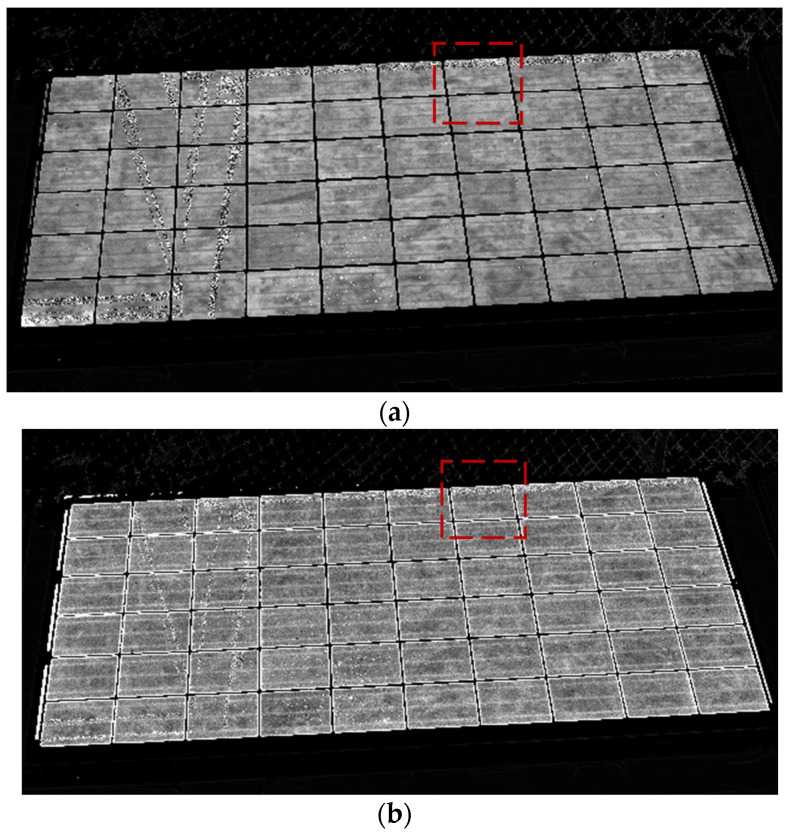
EL and PL images obtained using passive luminescence technique. (**a**) EL acquisition (Irradiance ≃ 800 W/m^2^ − exposure time = 4 ms − 200 cycles). (**b**) PL acquisition (Irradiance ≃ 800 W/m^2^ − exposure time = 4 ms − 200 cycles).

Figure 7 displays both EL (Figure 7a) and PL (Figure 7b) final pictures after processing of one PV module. It can be observed in both pictures that the luminescence signal intensity is uniformly spread across all the cells of the modules, indicating good health and performance of the module. The state of this module has also been measured through its current–voltage curve, which does not reveal any major failures. The parameters of the current–voltage curve, including V_OC_, V_MPP_, I_SC_, I_MPP_, and Power, are displayed in Table 2, showing a degradation, especially in the current, which can be considered normal as the module has been in operation for a few years. In this case, the luminescence pictures allow us to understand that the module degradation measured in the current–voltage curve is caused by uniform degradation and is not caused by major faults in certain solar cells.

Comparing EL with PL pictures, EL displays higher quality, which is probably due to the higher intensity of EL signal compared to PL signal.

**Figure 8:**
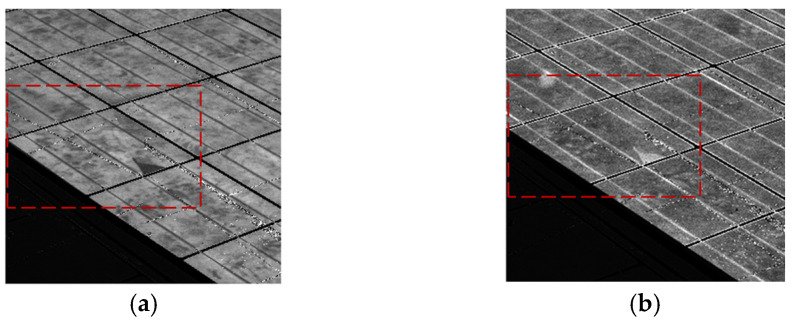
EL and PL images obtained using passive luminescence technique, which show a cell with a minor crack and inactive area. (**a**) EL acquisition (Irradiance ≃ 600 W/m^2^ − exposure time = 7 ms − 200 cycles). (**b**) PL acquisition (Irradiance ≃ 600 W/m^2^ − exposure time = 7 ms − 200 cycles).

Figure 8 displays closer EL and PL pictures of the same module, revealing a cell with a minor fault (that cell is surrounded by a red square in Figure 7 and Figure 8). It is possible to see how a crack has led to a small part of the solar cell area not being electrically connected. Therefore, the EL signal is null, as can be seen in Figure 8a. However, the PL signal in this area is high in Figure 8b as this part of the cell, although not connected with the rest of the cell and therefore current can be extracted or injected, the quality of the material is still good. In this way, EL and PL pictures provide complementary information on the module performance.

Another major issue is to analyze the behavior of the inverter during the measurement process. For PL acquisition, the behavior of the inverter is less critical as the tested module is bypassed. However, for EL acquisition, it is crucial that the inverter maintains a high current when the board changes the polarity of the module, since the current of the string is going to be injected into the tested module. Therefore, if the current drops to a value close to zero, EL measurement will be impossible to perform. Hence, the current and voltage of the string have been monitored during the measurement process for EL acquisition.

It should be noted that changing the polarity of the module will lead to a reduction in the voltage of the string because the module starts behaving as a load. It is essential that the new voltage of the string remains within the thresholds of the maximum power point tracking; otherwise, the current will drop to zero.

**Figure 9:**
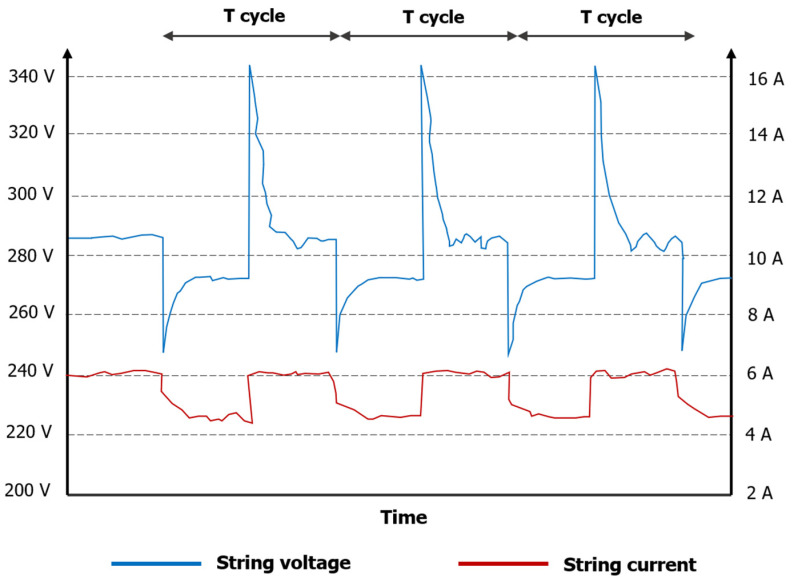
String current and voltage evaluation during EL measurement process.

Figure 9 displays the measured current and voltage of the PV string of the PV facility while the board changes one module’s state between MPP and CI for the EL acquisition process. Variations in both current and voltage may be appreciated. However, this variation does not affect the measurement process. The string current presents a small drop of around 15–20% compared to the current when the string is operating under normal conditions. Nonetheless, the string current remains high enough to produce an EL signal high enough for *lock-in* integration under high irradiance conditions. In addition, the string voltage never falls below the lower threshold of the maximum power point tracking system of the inverter (160 V for the inverter used in this experiment).

It is worth noting that most solar power plants feature larger photovoltaic strings, as well as a central inverter where one MPP tracking system is responsible for hundreds, even thousands of modules. Therefore, in these scenarios, it is expected that voltage and current variations would be lower, perhaps even imperceptible.

## 5. Discussion and Conclusions

The present work has demonstrated that integrating an electronic board into a PV module could enhance the acquisition of passive EL and PL pictures. Passive acquisition means that the pictures are acquired during the normal operation of a solar power plant, leading to a non-invasive technique. The electronic board can change the polarity of the module, allowing current to be injected into the module, achieving EL effect without the need for an external power supply. The board can also bypass the module and create an open circuit in the module to generate a PL signal using sunlight as an excitation source. Since the luminescence signal is lower than sunlight intensity, implementing a *lock-in* technique is required. While other techniques provide a means to acquire PL pictures during normal operation in high irradiance conditions, the present technique is the first one to enhance the acquisition of EL pictures during the normal operation of a PV power plant.

The unique selling point of this technique is the fact that an external energy source is not necessary to achieve the EL and PL effect, and acquisition can be performed during normal operation of the power plant. Moreover, acquiring both PL and EL pictures could be interesting as they provide similar but complementary information on module performance. Since the pictures are taken during operation, if each module incorporates the developed electronics, disconnection of the modules is not required, reducing energy losses and decreasing safety issues. In fact, integrating this electronic concept into the modules and implementing a proper communication system will enhance the integration of a UAV for image acquisition, resulting in higher throughput and lower costs.

The main disadvantage of the technique is that both EL and PL signals depend on irradiance. While the PL signal is directly related to irradiance, the EL signal mainly depends on the injected current into the module. If the irradiance is low, the current of the string will also be low, resulting in a low EL signal. Therefore, measurements are always limited by irradiance thresholds.

Future work will involve addressing several issues. Firstly, a power supply system will be developed to feed the board using the solar module voltage. This will eliminate the need for an external voltage supply for the board to function. Secondly, software improvements will enable the integration of a synchronization system between the board and the camera, as well as the processing algorithm. This integration will allow for the processing of pictures while they are being captured. Therefore, it will be possible to evaluate how many pictures and cycles are needed to achieve a minimum standard of quality. Finally, a communications module will be integrated into the board to send commands to the board through the DC power cable of the plant from the inverter. As it is expected that each module will have one electronic board, an identification system will also be implemented in order to send commands to selected modules. This improvement will enhance efficiency and throughput, resulting in cost reduction. Moreover, they also facilitate the integration of image acquisition with UAVs. Consequently, operators are not required, and measurements can be automatically conducted, saving both time and money.

## Figures and Tables

**Table 1:** Summary of new approaches for EL and PL imaging in field inspection of PV power plants.

Technique	EL/PL	Day/Night	*Lock-In*	Discussion
PL with artificial light [16]	PL	Night	No	Technique that requires a lighting system to achieve PL effect and an InGaAs camera. It enables contactless measurements during the night, eliminating the need for module disconnections and thereby increasing throughput.
PV mobile lab [17,18]	EL	Day/Night	No	PV modules are dismantled, and various measurements are conducted in a truck, including EL images captured with a silicon-based sensor camera in dark conditions. While measurements with laboratory standards are achieved, the disassembly of modules can be time-consuming and may lead to the generation of faults.
Bidirectional inverter [19]	EL	Night	No	Bidirectional inverters can be installed in PV power plants to enhance current injection into the PV array, generating the EL effect. Consequently, contactless EL images can be acquired during the night.
Daylight EL and PL [20]	EL/PL	Day	Yes	An electronic device, positioned between a power supply and the tested module, modulates an EL signal for *lock-in* technique integration. The electronic device can function without a power supply, modulating a PL signal using sunlight as an excitation source. While measurements during the day are feasible, module disconnection is necessary for this process.
Daylight PL (control cell method) [21,22,23]	PL	Day	Yes	Technique that utilizes sunlight as an excitation source and modulates a PL signal for *lock-in* integrations by controlling one cell per substring in a module. If the control cell is shaded, all the substrings operate in an open circuit condition. Conversely, if the cell is illuminated, the substring operates at the maximum power point.
Daylight PL (inverter control method) [24]	PL	Day	Yes	Technique that uses sunlight as excitation source and modulates a PL signal for *lock-in* integrations thanks to the control of the PV working point thought the inverter, which changes the state between maximum power point and open circuit. No additional hardware is required in comparison with other techniques.
Self-sourced EL [25]	EL	Day	Yes	Technique that uses an electronic device for modulating an EL signal for *lock-in* integrations. The device collects the energy generated by the tested module to store it, boost the voltage, and inject the current into the module achieving EL effect. EL measurements during the day are possible without the need for a power supply.
Daylight PL at constat operating point [26]	PL	Day	Yes	PL technique that uses sunlight as excitation source and allows *lock-in* integration at constant operational point. It can be possible because of the use of different filters that enhance acquiring images with low and high PL intensity.
Daylight PL with ultranarrow bandpass filter [27]	PL	Day	No	PL technique that uses sunlight as the excitation source without employing a *lock-in* technique. The incorporation of a custom ultranarrow bandpass filter enables the capture of the PL signal within a small wavelength gap where the solar signal is nearly negligible due to absorption by water vapor in the atmosphere.
PL system integrated in a cleaning robot [28]	PL	Day/Night	No	Cleaning robot designed for PV power plants incorporates a photoluminescence (PL) imaging acquisition system, featuring an LED lighting system and a silicon-based camera. The robot captures multiple images, which are then combined and reconstructed to cover the entire surface of the modules.

**Table 2:** Current–voltage curve parameters of the tested PV module.

Parameter	Measured (STC)	Datasheet (STC)	Difference
V_OC_	37.07 V	37.1 V	−0.08%
V_MPP_	28.57 V	30.1 V	−5.08%
I_SC_	7.59 A	8.31 A	−8.66%
I_MPP_	6.82 A	7.81 A	−12.68%
Power_MPP_	194.93 Wp	235 Wp	−17.05%

## Data Availability

No new data were created or analyzed in this study. Data sharing is not applicable to this article.
